# On Thoracic Percussion and Abdominal Compression

**Published:** 1823-12-01

**Authors:** 


### *Thoracic Percussion and Abdominal Pressure.**

Two distinct processes contribute to the advancement of practical medicine; reasoning and observation. The first, commonly exaggerated or premature, leads frequently to error; and hence doubtless it is that medicine has long been considered a conjectural science, destitute of certainty, and unworthy of credit: observation, on the other hand, an inexhaustible source of facts, presents invariably the image of truth—to those at least who are skilled in the inquiry.

Amid the numerous and infinitely varied derangements to which our frail organization is obnoxious, thoracic diseases have ever held a conspicuous rank, as well from their violence as from the frequent difficulty attendant on the discrimination of them. Hence the physicians of all ages have been anxiously employed in delineating their peculiar characters; and the sketch has been augmented or curtailed by successive observers, with the view of rendering it more unequivocal or concise.

The employment of percussion of the chest to elucidate

---

* Last edition of Desault's surgical works, by Roux.

the diagnosis of thoracic diseases, merits, amid these numerous efforts, particular notice. It was probably unknown to the ancients. This discovery, the honour of which is attributed to Awenbrugger,* a physician of Vienna, long slumbered in the oblivion, to which, on the decease of its author, it had been consigned; till, in our days, revived by Corvisart. The advantages which he has derived from it are universally known; in his hands, it has become signally instrumental in removing the dense veil with which diseases of the thorax, particuarly those of a chronic nature, have hitherto been obscured.

Bichat was admirably calculated by his genius to improve the science, upon which all the energies of his great mind were concentrated. From the dawn of his professional career, he sought, in treading the steps of his more illustrious predecessors, to establish, upon the results of observation and experience, the impregnable basis of his reputation. We are now contemplating his labours only as far as they regard our present subject. He confirmed many of the results obtained from percussion of the thorax: others assumed, in his view, a dubious aspect; and we shall presently see that he made a more correct and extensive application of them to the distinction of acute diseases.

The idea of abdominal pressure was suggested to him by a remark which physicians have long since made. It was observed that patients, suffering from hydrothorax, aneurism of the heart, and other organic lesions of the thorax, experienced a degree of uneasiness amounting to suffocation, whenever the stomach became distended. Hence this symptom was considered as one in the series, essentially attendant upon these diseases.

If any other circumstance contribute to produce this phenomenon, it must principally arise from the mechanical distention of the abdominal parietes, and from the contraction of the thorax by the elevation of the diaphragm. Now, to effect this elevation in a prompt manner, by compressing from below upwards the epigastrium or hypochondria, according to the seat of the affection which is the object of our research; to watch attentively the effect produced upon the patient; and to apply the result of this manual experiment, in order

---

* Awenbrugger published his discovery, and the results of his experience respecting its application, in a Latin work. Corvisart, some years since, promised a translation of it into French, illustrated by his own comments. We have long been inquiring for this publication, but cannot ascertain whether the design of the celebrated pathologist was ever carried into execution.

to acquire a certainty of the existence of any particular disease;—in this consists abdominal pressure, as a new mean of aiding the diagnosis of thoracic maladies.

Were no greater advantages than those resulting from percussion of the chest offered by this process, the publication of it would be by no means destitute of utility, and this Memoir might be entitled to attention; for in diseases thus obscure, and characterized only by the assemblage of a number of signs which are all uncertain when separately viewed, any new feature or mean of diagnosis, added to the sum of those already known, must be favourably received. But abdominal pressure furnishes, in many cases, results more certain than percussion: and again, this last is more generally applicable to the discrimination of diseases than has hitherto been supposed. The developement of these propositions constitutes the express object of our Memoir: in accomplishing it, we shall apply, in a comparative manner, these two methods to the investigation of the signs of acute and chronic affections of the chest. We begin with the first.

The interest which we feel in seizing the distinctive characters of the diseases affecting contiguous organs, when their difference of seat is not clearly established by the symptoms arising from them, can only be excited by the desire of classifying them in a rigid and precise manner, or by the necessity of instituting an important modification in their treatment. When these motives are combined, such anxiety is amply justified. This idea is naturally suggested by the numerous dissentions which have arisen upon the frequently agitated question—Exists there a pleurisy distinct from peripneumony? and, admitting that, can we by certain characters distinguish inflammation of the pleura from that of the lungs?

In the existing state of anatomical science, we can correctly appreciate all those reasonings, by the aid of which inflammations of the lungs, and of their membranous coverings, were, for a time, confounded in theory and practice, under the common idea of fluxion of the breast. We are now perfectly convinced, that organs, possessing a distinct structure and animated by a peculiar life, cannot correctly be confounded with regard to their morbid affections: and already many of those, who at first refused their assent to the preceding proposition, have relaxed from their severity in analogous cases, and been compelled, by the inspection of bodies, to acknowledge the existence of peritoneal inflammation distinct from that of the subjacent coats of the stomach, intestines, bladder, and hepatic structure; inflammation of the pericardium separate from that of the heart; and inflammation of the membranes of the brain, the substance of the latter organ remaining completely unaffected.

It must, however, be allowed, that, in the number of physicians who have pronounced a negative upon the foregoing question,* some have been influenced by the real or disputed observation of Servius; who says, that upon the dissection of three hundred subjects dying at Rome of thoracic inflammation, he had invariably found congestion of the lungs, the pleura exhibiting no trace of previous inflammation. But whoever reflects properly upon this remark, admitting it to be correct, must perceive, that, far from being favourable to the opinion of the physicians in question, it, on the contrary, proves, first, that the substance of the lungs may be separately inflamed; secondly, that this condition is much more dangerous, and more frequently fatal, than inflammation of the pleura, since Servius, in his numerous dissections, observed not one instance of the latter disease.

Some of the older pathologists, and, in our times, Professors Corvisart and Pinel, have admitted the existence of pleuritic inflammation distinct from peripneumony. Now, to demonstrate the influence which a correct knowledge of the diagnosis of these two diseases may exert upon the judgment of the physician, and upon his treatment of them, is, in our opinion, the best justification of their researches that can be advanced.

In the first place, peripneumony must necessarily be more destructive than pleuritic inflammation. In fact, even had the observation of Servius, before alluded to, been wanting, that reasoning by which we have already been taught to conceive the possibility of their isolated existence, would surely induce us to augur more unfavourably of a case in which the substance of the lungs themselves is immediately affected, than of that in which it suffers only in a secondary manner, and from contiguity, as must necessarily happen in inflammation of the pleura.

Secondly, if however, on one hand, peripneumony be more impetuous in its progress; if it alarm more by the vehemence of its symptoms; yet, on the other, pleurisy is more formidable in its consequences. Thus, while we frequently see patients carried off in a few days by the intensity of the inflammatory action of the first, the second rarely proves fatal but by the formation of pus, which is one of its immediate consequences; or by the chronic inflammation, which readily becomes established, and soon terminates in serous effusion.

* Sennertus, Regius, Boerhaave, Hoffman, Bonetus, Morgagni, Haller, Cullen, Portal, are among the more celebrated of these writers.

Thirdly, it will be readily perceived, that two diseases, thus distinct in situation, may admit of certain modifications in their treatment. For example, does not local abstraction of blood offer peculiar advantages as a remedy in inflammation of the pleura, since part of this membrane lines the thoracic parietes, and has, doubtless, intimate connexions with the common integument? Actuated by this idea, Bichat gave an almost exclusive preference to the application of leeches upon the painful part of the chest. Frequently, when the inflammation ran high, he, soon after the application of them, covered the seat of pain with a sinapism or blister. The success resulting from this practice confirmed his opinions upon the inutility of general bleeding in pleuritic inflammation. On the contrary, the situation of the lungs, too deep within the chest to receive the influence of topical remedies; the fear of their excessive and inevitable fatal congestion; their intimate connexion with the circulation of the great vessels;—all these considerations authorize general abstraction of blood in peripneumony, and even call for its repeated employment, with the observance, however, of the prudent caution of not carrying it too far, if we would avoid the just reproach of the celebrated Morgagni: *Jam dictum, nullâ re magis in peripneumoniâ accelerari mortem, quàm sputi suppressione. Hæc autem sæpe accidit propter intempestivas, præsertim in senibus, missiones sanguinis: quanquam plures sunt medici qui ægros ob id interimunt, quia nesciunt ipsi quiescere.*

Do not the preceding observations shew the importance of accurately investigating the diagnostic signs of pleuritic and pneumonic inflammation? Certainly. And now, in the next place, let us inquire what respective utility the two processes of percussion of the chest and abdominal pressure may offer to us in this investigation.

The first, which in this as in other cases, is performed with the extremities of the fingers of one hand united, produces upon the diseased side, in *peripneumony*, a sound, of the obscurity of which one may best jndge by comparing it to that which results from striking, in the same manner, the sound side of the thorax. *In pleurisy*, on the other hand, no difference can be perceived in the sound arising from percussion of the two thoracic cavities.

The absence of any sensible result from abdominal pressure exercised upon a patient, in whom the existence of pleurisy may be suspected, is contrasted, in a striking manner, with the involuntary cough, the profound oppression, and sense of suffocation; which are the sudden effects of this pressure exercised below the costal cartilages of the affected side, so as to elevate the diaphragm, upon a subject in whom the combination of many other symptoms indicates the existence of inflammation of the lungs.

If, then, we find, united in the same patient, on the one hand, a clear sound of the painful side of the chest with insensibility of abdominal pressure; and, on the other hand, smallness and rapidity of respiration;—increased intensity of pain upon strong expiration,* because then the pleura participates the dilation of the thoracic parietes;—impracticability of lying long upon the affected side, because, in this situation, the lung must necessarily press upon the inflamed pleura, and augment the pain;—and, lastly, increase of this pain upon tolerably firm pressure of the intercostal spaces;—pleuritic inflammation is with certainty indicated. The fatal issue and dissection of a case exhibiting all these characters, have frequently confirmed to Bichat the accuracy of the opinion which he had previously delivered respecting it. To these phenomena we may further add the character of the pain which, in pleurisy, is poignant or somewhat lancinating: for, if there have been a period in the progress of science, in which it appeared ridiculous to assign a distinct character to the pain in pleuritic and pneumonic inflammation, the more profound attention with which the laws of our organization have since been studied, permits us, in these more enlightened days, to cherish no doubt upon the subject. In truth, we now know that each organ has its particular modification of pain, just as, in the natural state, the vital properties by which it is animated, display a peculiar character.

Peripneumony, again, is signalized by the following phenomena. The patient makes great efforts of inspiration, in order to supply the suspended functions of one part of the lungs. Here also expiration is painful, because the parietes of the thorax, in contracting, compress the inflamed organ in a greater degree, as the preceding inspiration has been more deep. The patient cannot lie down upon the sound side: now this phenomenon is presented, whenever the respiratory process is diminished in one of the lungs, as well in the case under consideration, as in hydrothorax. The pain is ordinarily deep and obtuse. Lastly, from what has been already observed, the suffocation, resulting from abdominal pressure, coincides, in peripnenmony, with the obscure and dead sound obtained from percussion of the patient's chest.

It will be seen, that in presenting the sketch of the dis-

* This is, we imagine, a typographical inaccuracy.—It should have been written *inspiration*.

tinctive phenomena of two diseases, hitherto frequently confounded by physicians, we have solely kept in view their peculiar symptoms—in other words, those which necessarily result from the local affection. We call them peculiar or proper symptoms; because, in all diseases which have a determinate seat, this order of phenomena is first developed; and afterwards those resulting from the disturbance which is necessarily excited in neighbouring or contiguous organs. Thus, in pleurisy we have almost always cough and expectoration, simply mucous or streaked with blood. The hiccup and vomiting, the diarrhœa or costiveness, which arise in peritoneal inflammation, are again symptoms of this nature, dependant on the connexion of the inflamed membrane with the diaphragm, stomach and intestines. The same may be said of the phrenitis which induces delirium and other symptoms characterising disturbance of the cerebral functions. Moreover, local affections exert their sympathetic influence upon many of the principal organs remotely situated; and hence springs that series of general phenomena which we can only explain by referring them to those functions upon the disturbance of which they seem to depend. Thus, derangement of the digestive powers is announced by those gastric symptoms with which the principal phlegmasiæ, in their origin, are so frequently complicated: thus, the increased action of the heart, constituting the attendant fever, announces the influence which this organ receives from them: and the flushing of the cheeks, a phenomenon sufficiently common in peripneumony and phthisis, denotes some change in the capillary circulation. It is indeed proved by the momentary suppression or increase of some, and often many, of the exhalations and secretions in constitutional or local diseases, that these functions are influenced by the general commotions of the system. The same remark will apply, although less commonly, to the functions of animal life.

But all these symptoms, whether sympathetic or dependant upon the nearness or contiguity of organs in local diseases, are so variable, and present themselves under modifications so questionable, that no rigorous or precise data can be founded upon them. Much less can we avail ourselves of them to determine the character, or fix the diagnosis, of diseases; the distinct and isolated nature of which may be yet doubtful. Hence, I have ranged none of them among the characteristic phenomena of pleuritic or pulmonary inflammation. By Bichat, this distinction in the symptoms of diseases was first completely developed: others before him had but faintly and imperfectly surveyed this luminous doctrine. His ideas upon it were never made public. Although perhaps defective in clearness and precision, they are here communicated with fidelity.

The long details into which we have been involuntarily drawn respecting pleurisy and peripneumony terminate here. We were prompted to this digression by the desire of combining the principal facts not recorded in the works of Bichat, as far as they are connected with our present object.

Notwithstanding the judicious remarks of Senac, Vicq-D'Azyr, Cullen, Stoll, and others, inflammation of the serous membrane of the pericardium, denominated pericarditis, is yet a disease of character too uncertain and defective to invite discussion. Other circumstances, moreover, deter us from it. In the first place, Bichat possessed no opportunity of making any essential remark upon this disease. From his silence, it may even be doubted whether he had ever accurately observed its progress. Secondly, even supposing that a suspicion of its existence was excited by certain symptoms, we are by no means certain that any useful results, as regards the diagnosis, may be drawn from percussion of the chest and abdominal pressure.

Yet Bichat frequently observed the consequences of this inflammation; particularly the adhesions, more or less intimate, of the heart to the pericardium. These adhesions, of which the pathologist distinguishes several species, are analagous to those of the other serous membranes. Thus an opportunity was afforded him of verifying and confirming a remark, already made by several distinguished writers,* that these old and intimate adhesions may sometimes, by the restraint which they impose upon the actions of the heart, be mistaken for aneurisms of that organ.

By philosophic physicians, especially in later times, it has been admitted that a disease, however incurable in its nature, ought not the less to be well known and rigidly investigated: otherwise, in the uncertainty inseparable from confusion, we may direct against it remedies, which, although entitled to our confidence when judiciously employed, will, under opposite circumstances, prove pernicious to the patient. In this respect, medicine has indeed made, of late, considerable progress. Thus many forms of dropsy, so long regarded as diseases invariably idiopathic, and treated as such, have at last been found to result from internal changes of organic structure necessarily fatal, and to claim only the rank of secondary phenomena. Yet the very men who have acquired such honour in thus extending the circle of our knowledge,

---

* Lower, Vieussens, Senac, Morgagni, Vicq-D'Azyr.

have not neglected to investigate the signs by which, in difficult cases, these effusions may be distinguished. On the other hand, their efforts to attain this most essential object claim for them additional respect.

It is, nevertheless, necessary to guard against the baneful extremes into which by these views some practitioners have been led: we mean, the almost utter abandonment, or at least improper restriction, of the operations employed, formerly perhaps in too indiscriminate a manner, for the cure of various dropsies. Thus it is with all the great events and revolutions in medicine. Time alone teaches us to estimate properly their importance and extent, and apply correctly to practice the knowledge with which they furnish us.

Whether then it be our object merely to relieve for a time the sufferings of those doomed to inevitable death, and thus prolong existence to the utmost possible term, or to effect that which is but too rarely attained, a permanent cure, we may perhaps with advantage more frequently employ those operations destined to evacuate the serous or other effusions of the various cavities; and examples of the benefit resulting from them, when undertaken in the first view, are not wanting. Some author, whose name we do not recollect, mentions that a woman had thirty times undergone the operation for ascites. We remember, ourselves, to have seen it performed upon a lady, of twenty-six years, for the seventy-second time. She died on the ensuing day. The results of the dissection, time has effaced from our memory. Employed even as a mean of cure, the evacuation of the fluid of dropsy has not invariably failed of success. Fortunate cases of ascites, thus cured, are recorded by authors. We have seen a woman completely recovered after having eight times submitted to *paracentesis.* Examples of this success are, however, more rare with regard to hydrothorax. Yet there are some, the authenticity of which we cannot doubt. Among others, we may cite a very curious case communicated by Morand in the Memoirs of the Academy of Surgery.

Whatever then be the judgment which we form upon a case of effusion, and the treatment to be adopted, it is always necessary to distinguish correctly both its existence and its peculiar nature, in endeavouring to set up by nice observation a more complete assemblage of diagnostic phenomena.

The solidity of the parietes of the thorax, and their want of extensibility, are principal causes of the difficulty which we encounter in distinguishing the different collections, of which this cavity, as well as the pericardium, may be the seat. A penetrating and experienced eye may indeed, upon viewing an assemblage of indeterminate circumstances, recognize the existence of effusion; but the generality of physicians can only acquire the decisive knowledge of the case by reiterated observation. For the last, then, it is useful to trace the certain characters of thoracic effusion. Before proceeding, we may remark that, although we treat here only of those spontaneous collections of which the pleura is susceptible, the ensuing observations may admit also of being applied to cases of sanguineous effusions of the chest, consequent upon the action of external violence.

Be it here observed, that the effusions which may take place in the pleura, do not all depend upon the same antecedent circumstances. Sometimes, for example, they succeed to pleurisy. In this case, the white flaky fluid is the product of the mode of suppuration, peculiar to serous membranes. Sometimes, a liquid is found somewhat different from the preceding; which may have its source either in chronic inflammation of the pleura, or result from a miliary eruption; a kind of morbid affection peculiar to these membranes; first slightly noticed by Morgagni, but since more frequently remarked by Bichat. Lastly, in the more common cases, hydrothorax, formed by a limpid and transparent fluid, is a phenomenon consequent upon the lesion of some neighbouring organ, and most frequently of the lungs or heart.

Although by authors a great number of signs, diagnostic of thoracic effusion, has been indicated; many of them are so uncertain that it would be difficult to found upon them exclusively, a decided opinion. It would be superfluous to repeat here what has been said, written, and repeated on this subject. We shall, therefore, claim attention only to three principal circumstances; which, when combined in a case, undoubtedly characterise the existence of thoracic effusion.

First. The patient can only lie down upon the side where the effusion exists; and the reason of this we indicated when speaking of peripneumonia (p. 574). It is not because, in the opposite position, the effused fluid presses upon the mediastinum and thus contracts the sound cavity, as some have pretended. Still, this circumstance may contribute some little, although not in an absolute manner, to the production of this phenomenon.

Secondly. Percussion of the chest may offer, in this case, great advantages. The obscurity of sound which we obtain from this process, is indeed remarkable: and the extent of this obscurity will be proportionate to the quantity of accumulated fluid. In order to appreciate this circumstance correctly, and to exercise the percussion with success, it is necessary that the patient should be seated. The fluid is then contained in the most depending part of the cavity.

There, consequently, we must strike the chest upon its anterior, posterior, and lateral aspects, listening attentively to the sound, and comparing it with that obtained by percussion of the other side of the chest. When the effusion is considerable, not only the result obtained is certain, but may be obtained to a very elevated part of the thoracic region. If, however, there be but little fluid in the pleura, the sound obtained may not be sufficiently obscure to admit of distinction from the natural state, or afford a sufficient ground of diagnosis. This inefficiency of percussion, in certain cases, joined to the fatigue which the patient experiences from the process, renders it a not invariably decisive means of diagnosis, especially in inexperienced hands: for it is a manœuvre which requires for its due performance a certain degree of practice. To this we may add, that with individuals of a corpulent habit, as with those of the female sex, the soft parts, covering the osseous parietes of the chest, absorb, in great part, sometimes even altogether, the strokes which it is our object to direct upon the latter.

Thirdly. If to the two phenomena just indicated, are united general agitation, cough, a sense of suffocation more or less considerable; originating from abdominal pressure exercised below the ribs of that side where the effusion is suspected to exist, so as absolutely to contract the cavity of the chest by an elevation of the diaphragm; we shall have acquired a certainty of the presence of fluid. And this will be especially the case, if we have duly acquainted ourselves with the circumstances which preceded the effusion, and the morbid affections to which the patient has been, and again may be, obnoxious. Any doubt upon the subject will still farther be diminished, if, notwithstanding the extremely equivocal character of the general symptoms, we find complicated with them habitual cough, smallness and concentration of the pulse, occasional syncope, and a lightly œdematous state of the integuments of the chest.

It may be suspected that, prejudiced in favour of abdominal pressure, we here give it an exclusive preference to percussion of the thorax. It is not so. We, on this subject, are delivering the ideas of Bichat rather than our own. His opinions were: first, that the former process possesses a decided superiority to the latter in certain cases where, as we have before observed, percussion, far from affording satisfactory results, may, on the other hand, mislead: secondly, that in other cases, where abdominal pressure does not offer the same advantages as percussion of the chest, it may yet always be regarded as a valuable resource in addition to the few we already possess: thirdly, that under some circumstances, it is not exempt from inconveniences; the knowledge of which it is important to acquire. For example, when the abdomen is greatly distended by a co-existent ascites, or by an effusion of serum into the cellular membrane of its parietes, pressure on this region may afford very feeble diagnostic light; and sometimes no accurate conclusion can be drawn from it. In some cases, the quantity of fluid effused into the chest may be so considerable, that by depression of the diaphragm, the liver is made to project considerably; and hence the complaints of a patient, thus circumstanced, upon pressure of the right hypochondrium, may be such as to induce a suspicion that the hepatic organ is diseased; and divert the attention of the physician from the real malady. Bichat had once, in such a case, the satisfaction of proving, by evacuation of the fluid collected in the chest, the accuracy of an opinion previously delivered by him, but contradicted by a celebrated man; who, upon an incautious examination, had declared that the liver was the seat of the disease.

One way of concurring essentially to the progress of medicine is, rather to avow the different sources of error and difficulty, than conceal them. Thus the attention of the observer will be kept upon the alert even under circumstances the least uncertain. This truth should be yet more forcibly inculcated with respect to the extensive class of organic affections. Although reduced, as they now are, to certain well-known species in each organ or system of organs, they assume, nevertheless, such different forms, and shew themselves under so many dubious aspects, that we ought constantly to be on our guard in order to avoid those mistakes in which the obscurity, reigning over them, may involve us. Thus all the characters of thoracic effusion may exist with diseases of a completely opposite nature. Corsivart relates a case in which, upon the examination of the subject, who was supposed to have died of effusion into the chest, one of the sides of that cavity was found filled with a very dense substance, of new formation, and every where adherent to its parietes: Every vestige of the lung had disappeared.

Other chronic affections of the lungs, as phthisis and inveterate catarrh, do not admit of the application of abdominal pressure or percussion of the chest. We, therefore, proceed to consider the last order of diseases; for the discrimination of which, experience has shewn that these two processes may be usefully employed,—pericardiac dropsy and aneurism of the heart.

The first of these affections commonly originates from the same causes which induce hydrothorax: they are indeed common to all the serous membranes. But all the signs, hitherto pointed out as characteristic of it, have served only to deceive even the most judicious and profound observers. No physician, even at the present time, and with a combination of the greatest number of probable signs, will decidedly pledge his opinion upon the existence of pericardiac effusion. The difficulty of establishing certain rules of diagnosis in this case, ought not to surprize us. Deeply situated, and rendered by the solidity of the neighbouring parts, almost inaccessible to our scrutiny, the pericardium is yet farther removed than the pleura from every mean of investigation, except abdominal pressure.

On the other hand, almost all the symptoms, enumerated as diagnostic of this malady, belong equally to disturbance of the functions of the heart, from whatever cause originating; and these causes may readily be confounded in practice. Yet, one important remark may here be made: no *permanent* and *uninterrupted* disturbance can exist in any viscus, independently on organic lesion, unless it be determined by some constitutional peculiarity of the individual in whom it occurs. In truth, every derangement of the functions of the heart, arising from a remote cause, is transitory as the influence of that cause by which it has been determined. It remains for us then to inquire, when the existence of organic disease is suspected, whether such disease be pericardiac dropsy or aneurism of the heart.

In the first place, it sometimes, although perhaps rarely happens, that the two diseases are complicated. Under these circumstances, we must direct our attention to the principal malady: It may be difficult to distinguish the secondary affection.

But more frequently, the substance of the heart itself being free from disease, the pericardium is filled consecutively with respect to the affection of some remote organ; as, for instance, one of the abdominal viscera. At other times, the effusion is preceded by symptoms of pericarditis. Again, the serous accumulation may, in certain cases, depend upon a peculiar affection of the pericardiac membrane, as a miliary eruption.

Under any of these three last circumstances, it becomes most important accurately to distinguish dropsy of the pericardium. Of the uncertainty of the signs, generally supposed to indicate it, we cannot be more deeply convinced than by meditating upon the two cases which Desault has recorded. In one of these, the symptoms, experienced by the patient, induced a suspicion of pericardiac effusion; and on the death of the subject, which followed the evacuation of the fluid by Desault, an extensive cyst was found adhering to the pericardium. In the other case, pericardiac dropsy was not detected till the decease of the patient, who was destroyed by an adventitious malady.

Percussion of the chest is not applicable to these cases; since, even in the natural state, the region occupied by the heart and its membranes, yields an obscure sound. Nevertheless, should the pericardium be very greatly dilated by the effused fluid, some opinion may be formed, from the unusual extent of this obscurity. Here then abdominal pressure claims a decided superiority; and, upon the *epigastrium*, it will be most effectually exercised. In that situation, the pericardium adheres to the tendinous centre of the diaphragm; and is consequently very near the parietes of the abdomen. Pressure also is more readily practised here than on the hypochondriac regions, on account of the hollow presented by the base of the thorax. Executed upon the principles already indicated, it augments in pericardiac dropsy, as in some other affections of the breast, the dyspnœa and sense of suffocation; and more especially denotes the disease by inducing sudden agitation of the heart and remote arteries and sometimes syncope. These phenomena subside or re-appear as the pressure is discontinued or renewed.

In all these researches, an accurate acquaintance with the circumstances which have preceded the actual state of the patient is necessary. A woman had an affection of the spleen, consequent upon a quartan intermittent fever. The abdomen swelled; and dubious symptoms of pericardiac effusion were soon manifested. Bichat practised abdominal pressure; and assured himself of the existence of that malady. The death of the patient enabled him to confirm, beyond all doubt, this opinion.

It seems that Bichat frequently preferred abdominal pressure to percussion of the chest, as an instrument of diagnosis in diseases of the heart Yet it must be allowed, that neither of these processes can be so usefully applied to such diseases as to the elucidation of certain other cases in which we have considered their employment and relative advantages. As auxiliaries to the precise means of discrimination in cardiac maladies which we, of late years, have acquired, they are, however, entitled to our notice.

In dilatation of the heart, we obtain by percussion an extremely obscure sound of the chest, corresponding in extent to the degree of dilatation. The certainty of this result, in most cases, cannot be denied. Yet Bichat had remarked that, in some patients exhibiting all the characters of *considerable* dilatation, the obscurity of sound was limited to a very *narrow* space; and this unusual result, he suspected, might be caused by the application of a portion of the lung upon the anterior surface of the pericardium. Hence, he was led to combine abdominal pressure with percussion of the chest; or entirely substitute the former for the latter. Pressure, however, can in this way be employed with advantage only when the dilated heart has acquired a certain volume.

But if it be exercised under circumstances favorable to its application, and in the manner as directed in cases of pericardiac dropsy, the patient experiences a distress resembling that which results from the horizontal posture. The suffocation, and lividity of the lips and countenance, increase in proportion to the degree of pressure ; the contractions of the heart become stronger : Indeed, such is the state of uneasiness and restraint suffered by patients, as well with diseased heart as with thoracic effusion upon whom this pressure is practised, that they all dreaded the visit of Bichat, apprehensive that he might torture them by a repetition of the experiment.

In closing our translation of this Memoir, we cannot but express a hope, that the British reader may glean from it some practical hints of value. Yet, favorable as we, on the whole, are induced to think of the processes therein recommended for elucidating the diagnosis of certain thoracic maladies, truth claims the acknowledgment, that we have not been able to derive from them all those decisive results of which they seem to have been productive in the hands of the more fortunate continental physicians.

